# Report of Deaths in Buffalo for the Month of November, 1855

**Published:** 1856-01

**Authors:** J. Root

**Affiliations:** Health Physician


ART. X.—*Report of Deaths in Buffalo for the month*

| DISEASES. | Males. | Females. | Total. | AGE. 1 year and Under. Male. | Female. | 1 to 2 years. Male. | Female. |
|---|---|---|---|---|---|---|---|
| Anasarca, | 1 | .... | 1 | .. | .. | .. | .. |
| Apoplexy, | .... | 2 | 2 | .. | .. | .. | .. |
| Atrophia, | .... | 1 | 1 | .. | .. | .. | .. |
| Angina, | .... | 1 | 1 | .. | .. | .. | .. |
| Asthma, | 1 | 1 | 2 | .. | .. | .. | .. |
| Bronchitis, | 1 | .... | 1 | .. | .. | .. | .. |
| Consumption, | 16 | 6 | 22 | 3 | 3 | 2 | .. |
| Convulsions, | 6 | 3 | 9 | 6 | 2 | .. | 1 |
| Croup Membranous, | 2 | 1 | 3 | .. | .. | 1 | 1 |
| Croup, | 3 | .... | 3 | 1 | .. | 1 | .. |
| Cancer of Stomach, | .... | 1 | 1 | .. | .. | .. | .. |
| " of Penis, | 1 | .... | 1 | .. | .. | .. | .. |
| Congestion of Lungs, | .... | 1 | 1 | .. | 1 | .. | .. |
| Cholera Infantum, | .... | 1 | 1 | .. | 1 | .. | .. |
| Disease of Heart, | 1 | .... | 1 | .. | .. | .. | .. |
| Drowned, | 2 | .... | 2 | .. | .. | .. | .. |
| Dropsy of Brain, | 1 | .... | 1 | .. | .. | .. | .. |
| " of Heart, | .... | 1 | 1 | .. | .. | .. | .. |
| Debility, | .... | 1 | 1 | .. | 1 | .. | .. |
| Delirium Tremens, | 1 | .... | 1 | .. | .. | .. | .. |
| Dysentery, | .... | 1 | 1 | .. | .. | .. | .. |
| Dentition, | 1 | 1 | 2 | 1 | .. | .. | 1 |
| Diarrhœa, | 1 | .... | 1 | .. | .. | .. | .. |
| Erysipelas, | 1 | 1 | 2 | .. | 1 | .. | .. |
| Epilepsy, | 1 | .... | 1 | .. | .. | .. | .. |
| Fever Typhoid, | 4 | 2 | 6 | .. | .. | .. | .. |
| " Typhus, | 1 | .... | 1 | .. | .. | .. | .. |
| " Scarlet, | 4 | 6 | 10 | .. | .. | 2 | .. |
| " Brain, | .... | 1 | 1 | .. | .. | .. | .. |
| "Febris Nervosa," | .... | 1 | 1 | .. | .. | .. | .. |
| Fracture of Cranium, | 1 | .... | 1 | .. | .. | .. | .. |
| Hæmorrhage from Lungs, | 1 | .... | 1 | .. | .. | .. | .. |
| Inflammation of Brain, | 1 | 1 | 2 | .. | .. | .. | .. |
| Inflammation, | 1 | .... | 1 | 1 | .. | .. | .. |
| Laryngitis, | .... | 1 | 1 | .. | .. | .. | .. |
| Measles, | 1 | .... | 1 | .. | .. | 1 | .. |
| Old Age, | 1 | 1 | 2 | .. | .. | .. | .. |
| Pneumonia, | .... | 3 | 3 | .. | 1 | .. | .. |
| Premature Birth, | 1 | .... | 1 | 1 | .. | .. | .. |
| Pleurisy, | .... | 1 | 1 | .. | 1 | .. | .. |
| Suicide, | 1 | .... | 1 | .. | .. | .. | .. |
| Still-born, | 1 | 3 | 4 | 1 | 3 | .. | .. |
| Suffocation, | 1 | .... | 1 | 1 | .. | .. | .. |
| Unknown, | 8 | 1 | 9 | 3 | 1 | 2 | .. |
| Whooping Cough, | 3 | 3 | 6 | 2 | 1 | 1 | 2 |
| | | | | .. | .. | .. | .. |
| | | | | .. | .. | .. | .. |
| | | | | .. | .. | .. | .. |
| | | | | .. | .. | .. | .. |
| | | | | .. | .. | .. | .. |
| | | | | .. | .. | .. | .. |
| Totals, | 70 | 47 | 117 | 20 | 16 | 10 | 5 |

NATIVITIES.—American, 48. German, 38. Irish, 15. Scotch, 1. French, 3.

*of November*, 1855. By J. Root, M. D., Health Physician.

AGE.

| 2—5 | | 5—10 | | 10—15 | | 15—20 | | 20—25 | | 25—30 | | 30—40 | | 40—50 | | 50—60 | | 60—70 | | 70—80 | | 80—90 | | 90—100 | | 100 and upward. | | Not given. | |
|---|---|---|---|---|---|---|---|---|---|---|---|---|---|---|---|---|---|---|---|---|---|---|---|---|---|---|---|---|---|
| Male. | Female. | Male. | Female. | Male. | Female. | Male. | Female. | Male. | Female. | Male. | Female. | Male. | Female. | Male. | Female. | Male. | Female. | Male. | Female. | Male. | Female. | Male. | Female. | Male. | Female. | Male. | Female. | Male. | Female. |
| .. | .. | .. | .. | 1 | .. | .. | .. | .. | .. | .. | .. | .. | .. | .. | .. | .. | .. | .. | .. | .. | .. | .. | .. | .. | .. | .. | .. | .. | .. |
| .. | .. | .. | .. | .. | .. | .. | .. | .. | .. | .. | .. | .. | 1 | .. | .. | .. | .. | .. | .. | .. | .. | .. | 1 | .. | .. | .. | .. | .. | .. |
| .. | 1 | .. | .. | .. | .. | .. | .. | .. | .. | .. | .. | .. | .. | .. | .. | .. | .. | .. | .. | .. | .. | .. | .. | .. | .. | .. | .. | .. | .. |
| .. | .. | .. | .. | .. | 1 | .. | .. | .. | .. | .. | .. | .. | .. | .. | .. | .. | .. | .. | .. | .. | .. | .. | .. | .. | .. | .. | .. | .. | .. |
| .. | .. | .. | .. | .. | .. | .. | .. | .. | .. | .. | .. | .. | .. | .. | 1 | .. | .. | 1 | .. | .. | .. | .. | .. | .. | .. | .. | .. | .. | .. |
| 1 | .. | .. | .. | .. | .. | .. | .. | .. | .. | .. | .. | .. | .. | .. | .. | .. | .. | .. | .. | .. | .. | .. | .. | .. | .. | .. | .. | .. | .. |
| 1 | 1 | .. | .. | 1 | .. | .. | .. | 1 | .. | 5 | 1 | 2 | 1 | 1 | .. | .. | .. | .. | .. | .. | .. | .. | .. | .. | .. | .. | .. | .. | .. |
| .. | .. | .. | .. | .. | .. | .. | .. | .. | .. | .. | .. | .. | .. | .. | .. | .. | .. | .. | .. | .. | .. | .. | .. | .. | .. | .. | .. | .. | .. |
| .. | .. | 1 | .. | .. | .. | .. | .. | .. | .. | .. | .. | .. | .. | .. | .. | .. | .. | .. | .. | .. | .. | .. | .. | .. | .. | .. | .. | .. | .. |
| 1 | .. | .. | .. | .. | .. | .. | .. | .. | .. | .. | .. | .. | .. | .. | .. | .. | .. | .. | .. | .. | .. | .. | .. | .. | .. | .. | .. | .. | .. |
| .. | .. | .. | .. | .. | .. | .. | .. | .. | .. | .. | .. | .. | .. | .. | 1 | .. | .. | .. | .. | .. | .. | .. | .. | .. | .. | .. | .. | .. | .. |
| .. | .. | .. | .. | .. | .. | .. | .. | .. | .. | .. | .. | .. | .. | .. | .. | .. | .. | 1 | .. | .. | .. | .. | .. | .. | .. | .. | .. | .. | .. |
| .. | .. | .. | .. | .. | .. | .. | .. | .. | .. | .. | .. | .. | .. | .. | .. | .. | .. | .. | .. | .. | .. | .. | .. | .. | .. | .. | .. | .. | .. |
| .. | .. | .. | .. | .. | .. | .. | .. | .. | .. | .. | .. | .. | .. | .. | .. | .. | .. | .. | .. | .. | .. | .. | .. | .. | .. | .. | .. | .. | .. |
| .. | .. | .. | .. | .. | .. | .. | .. | .. | .. | .. | .. | .. | .. | .. | .. | 1 | .. | .. | .. | .. | .. | .. | .. | .. | .. | .. | .. | .. | .. |
| .. | .. | .. | .. | .. | .. | .. | .. | .. | .. | 1 | .. | 1 | .. | .. | .. | .. | .. | .. | .. | .. | .. | .. | .. | .. | .. | .. | .. | .. | .. |
| 1 | .. | .. | .. | .. | .. | .. | .. | .. | .. | .. | .. | .. | .. | .. | .. | .. | .. | .. | .. | .. | .. | .. | .. | .. | .. | .. | .. | .. | .. |
| .. | .. | .. | 1 | .. | .. | .. | .. | .. | .. | .. | .. | .. | .. | .. | .. | .. | .. | .. | .. | .. | .. | .. | .. | .. | .. | .. | .. | .. | .. |
| .. | .. | .. | .. | .. | .. | .. | .. | .. | .. | .. | .. | .. | .. | .. | .. | .. | .. | .. | .. | .. | .. | .. | .. | .. | .. | .. | .. | .. | .. |
| .. | .. | .. | .. | .. | .. | .. | .. | .. | .. | .. | .. | .. | .. | 1 | .. | .. | .. | .. | .. | .. | .. | .. | .. | .. | .. | .. | .. | .. | .. |
| .. | .. | .. | .. | .. | .. | .. | .. | .. | .. | .. | .. | .. | .. | .. | .. | .. | 1 | .. | .. | .. | .. | .. | .. | .. | .. | .. | .. | .. | .. |
| .. | .. | .. | .. | .. | .. | .. | .. | .. | .. | .. | .. | .. | .. | .. | .. | .. | .. | .. | .. | .. | .. | .. | .. | .. | .. | .. | .. | .. | .. |
| 1 | .. | .. | .. | .. | .. | .. | .. | .. | .. | .. | .. | .. | .. | .. | .. | .. | .. | .. | .. | .. | .. | .. | .. | .. | .. | .. | .. | .. | .. |
| .. | .. | .. | .. | .. | .. | 1 | .. | .. | .. | .. | .. | .. | .. | .. | .. | .. | .. | .. | .. | .. | .. | .. | .. | .. | .. | .. | .. | .. | .. |
| .. | .. | .. | .. | .. | .. | .. | .. | .. | .. | .. | .. | .. | .. | .. | .. | .. | .. | 1 | .. | .. | .. | .. | .. | .. | .. | .. | .. | .. | .. |
| .. | .. | .. | .. | 1 | .. | 2 | .. | .. | 1 | .. | .. | 1 | .. | .. | .. | .. | .. | .. | .. | .. | .. | .. | .. | .. | .. | .. | .. | .. | 1 |
| .. | .. | .. | .. | .. | .. | 1 | .. | .. | .. | .. | .. | .. | .. | .. | .. | .. | .. | .. | .. | .. | .. | .. | .. | .. | .. | .. | .. | .. | .. |
| 1 | 3 | .. | 2 | 1 | 1 | .. | .. | .. | .. | .. | .. | .. | .. | .. | .. | .. | .. | .. | .. | .. | .. | .. | .. | .. | .. | .. | .. | .. | .. |
| .. | 1 | .. | .. | .. | .. | .. | .. | .. | .. | .. | .. | .. | .. | .. | .. | .. | .. | .. | .. | .. | .. | .. | .. | .. | .. | .. | .. | .. | .. |
| .. | .. | .. | .. | .. | .. | .. | .. | .. | .. | .. | .. | .. | 1 | .. | .. | .. | .. | .. | .. | .. | .. | .. | .. | .. | .. | .. | .. | .. | .. |
| .. | .. | .. | .. | .. | .. | .. | .. | .. | .. | .. | .. | .. | .. | .. | .. | 1 | .. | .. | .. | .. | .. | .. | .. | .. | .. | .. | .. | .. | .. |
| .. | .. | .. | .. | .. | .. | .. | .. | .. | .. | .. | .. | 1 | .. | .. | .. | .. | .. | .. | .. | .. | .. | .. | .. | .. | .. | .. | .. | .. | .. |
| 1 | 1 | .. | .. | .. | .. | .. | .. | .. | .. | .. | .. | .. | .. | .. | .. | .. | .. | .. | .. | .. | .. | .. | .. | .. | .. | .. | .. | .. | .. |
| .. | .. | .. | .. | .. | .. | .. | .. | .. | .. | .. | .. | .. | .. | .. | .. | .. | .. | .. | .. | .. | .. | .. | .. | .. | .. | .. | .. | .. | .. |
| .. | 1 | .. | .. | .. | .. | .. | .. | .. | .. | .. | .. | .. | .. | .. | .. | .. | .. | .. | .. | .. | .. | .. | .. | .. | .. | .. | .. | .. | .. |
| .. | .. | .. | .. | .. | .. | .. | .. | .. | .. | .. | .. | .. | .. | .. | .. | .. | .. | .. | .. | .. | .. | .. | .. | .. | .. | .. | .. | .. | .. |
| .. | .. | .. | .. | .. | .. | .. | .. | .. | .. | .. | .. | .. | .. | .. | .. | .. | .. | 1 | 1 | .. | .. | .. | .. | .. | .. | .. | .. | .. | .. |
| .. | 1 | .. | 1 | .. | .. | .. | .. | .. | .. | .. | .. | .. | .. | .. | .. | .. | .. | .. | .. | .. | .. | .. | .. | .. | .. | .. | .. | .. | .. |
| .. | .. | .. | .. | .. | .. | .. | .. | .. | .. | .. | .. | .. | .. | .. | .. | .. | .. | .. | .. | .. | .. | .. | .. | .. | .. | .. | .. | .. | .. |
| .. | .. | .. | .. | .. | .. | .. | .. | .. | .. | .. | .. | .. | .. | .. | .. | .. | .. | .. | .. | .. | .. | .. | .. | .. | .. | .. | .. | .. | .. |
| .. | .. | .. | .. | .. | .. | .. | .. | .. | .. | .. | .. | 1 | .. | .. | .. | .. | .. | .. | .. | .. | .. | .. | .. | .. | .. | .. | .. | .. | .. |
| .. | .. | .. | .. | .. | .. | .. | .. | .. | .. | .. | .. | .. | .. | .. | .. | .. | .. | .. | .. | .. | .. | .. | .. | .. | .. | .. | .. | .. | .. |
| .. | .. | .. | .. | .. | .. | .. | .. | .. | .. | .. | .. | .. | .. | .. | .. | .. | .. | .. | .. | .. | .. | .. | .. | .. | .. | .. | .. | .. | .. |
| .. | .. | .. | .. | .. | .. | 1 | .. | .. | .. | .. | .. | .. | .. | 1 | .. | 1 | .. | .. | .. | .. | .. | .. | .. | .. | .. | .. | .. | .. | .. |
| .. | .. | .. | .. | .. | .. | .. | .. | .. | .. | .. | .. | .. | .. | .. | .. | .. | .. | .. | .. | .. | .. | .. | .. | .. | .. | .. | .. | .. | .. |
| .. | .. | .. | .. | .. | .. | .. | .. | .. | .. | .. | .. | .. | .. | .. | .. | .. | .. | .. | .. | .. | .. | .. | .. | .. | .. | .. | .. | .. | .. |
| .. | .. | .. | .. | .. | .. | .. | .. | .. | .. | .. | .. | .. | .. | .. | .. | .. | .. | .. | .. | .. | .. | .. | .. | .. | .. | .. | .. | .. | .. |
| .. | .. | .. | .. | .. | .. | .. | .. | .. | .. | .. | .. | .. | .. | .. | .. | .. | .. | .. | .. | .. | .. | .. | .. | .. | .. | .. | .. | .. | .. |
| .. | .. | .. | .. | .. | .. | .. | .. | .. | .. | .. | .. | .. | .. | .. | .. | .. | .. | .. | .. | .. | .. | .. | .. | .. | .. | .. | .. | .. | .. |
| .. | .. | .. | .. | .. | .. | .. | .. | .. | .. | .. | .. | .. | .. | .. | .. | .. | .. | .. | .. | .. | .. | .. | .. | .. | .. | .. | .. | .. | .. |
| .. | .. | .. | .. | .. | .. | .. | .. | .. | .. | .. | .. | .. | .. | .. | .. | .. | .. | .. | .. | .. | .. | .. | .. | .. | .. | .. | .. | .. | .. |
| 7 | 9 | 1 | 4 | 4 | 2 | 5 | 0 | 1 | 1 | 6 | 1 | 6 | 3 | 3 | 2 | 3 | 1 | 4 | 1 | 0 | 0 | 0 | 1 | 0 | 0 | 0 | 0 | 0 | 1 |

Hollander, 1. Welch, 1. Colored, 2. Country not given, 8.—Total, 117.

